# Visualizing changes in brain-derived neurotrophic factor (BDNF) expression using bioluminescence imaging in living mice

**DOI:** 10.1038/s41598-017-05297-x

**Published:** 2017-07-10

**Authors:** Mamoru Fukuchi, Hironori Izumi, Hisashi Mori, Masahiro Kiyama, Satoshi Otsuka, Shojiro Maki, Yosuke Maehata, Akiko Tabuchi, Masaaki Tsuda

**Affiliations:** 1 Laboratory of Molecular Neuroscience, Faculty of Pharmacy, Takasaki University of Health and Welfare, 60 Nakaorui-machi, Takasaki-shi, Gunma 370-0033 Japan; 2 Department of Molecular Neuroscience, Graduate School of Medicine and Pharmaceutical Sciences, University of Toyama, 2630 Sugitani, Toyama-shi, Toyama 930-0194 Japan; 3 Department of Engineering Science, Graduate School of Informatics and Engineering, The University of Electro-Communications, 1-5-1 Chofugaoka, Chofu-shi, Tokyo 182-8585 Japan; 4 Department of Biological Chemistry, Graduate School of Medicine and Pharmaceutical Sciences, University of Toyama, 2630 Sugitani, Toyama-shi, Toyama 930-0194 Japan

## Abstract

Brain-derived neurotrophic factor (BDNF) plays a fundamental role in expressing various neural functions including memory consolidation. Alterations of BDNF levels in the brain are associated with neurodegenerative and neuropsychiatric disorders. Therefore, it is important to understand how levels of BDNF are controlled. Recently we generated a novel transgenic mouse strain, termed the *Bdnf*-*Luciferase* transgenic *(Bdnf*-*Luc* Tg) mouse, to monitor changes in *Bdnf* expression. In the present study, we detected the bioluminescence signal from living *Bdnf*-*Luc* Tg mice after intraperitoneal administration of d-luciferin. Despite high levels of *Bdnf* expression in the brain, it was difficult to detect a signal from the brain region, probably because of its poorly penetrable (short-wavelength) bioluminescence. However, we could detect the changes in the bioluminescence signal in the brain region using a luciferin analogue generating a near-infrared wavelength of bioluminescence. We also found a strong correlation between increases in body weight and bioluminescence signal in the abdominal region of Tg mice fed a high-fat diet. These results show that changes in *Bdnf* expression can be visualized using living mice, and that the Tg mouse could be a powerful tool for clarification of the role of *Bdnf* expression in pathophysiological and physiological conditions.

## Introduction

Brain-derived neurotrophic factor (BDNF) is a member of the neurotrophin family and contributes to expression of a variety of neural functions, such as neuronal survival, differentiation, plasticity, learning, and memory^1–4^. Because of its fundamental roles in neural functions, alterations of BDNF levels have been reported in neurodegenerative and neuropsychiatric disorders. For example, reduced BDNF expression has been observed in Alzheimer’s disease^5^, Parkinson’s diseases^6^, and Huntington’s disease^7^. Additionally, it has been reported that BDNF inducers or an agonist of the BDNF receptor, tropomyosin-related kinase B (TrkB), improved symptoms in model mice for neural disorders and stressed animals^8–11^. These reports strongly support the concept that BDNF is a biomarker and drug target for these diseases. Thus, it is important to understand alterations of BDNF levels in physiological and pathophysiological conditions.

In this study, we attempted to monitor the change in *Bdnf* expression *in vivo*. Imaging probes are very useful tools to approach this problem. One candidate for an imaging probe is a fluorescence probe using a fluorescent protein such as green fluorescent protein (GFP). Previously, the regulation of human *BDNF* expression was investigated by enhanced-GFP (EGFP)-based *in vivo* analysis using the bacterial artificial chromosome (BAC) or the yeast artificial chromosome (YAC) clone containing human *BDNF* ^12, 13^. In these reports, the expression of BDNF in brain sections prepared from transgenic mice was examined, and the results failed to demonstrate that changes in BDNF levels could be visualized in living mice. Furthermore, although fluorescence imaging brings about brighter and higher-resolution signals with autofluorescence noise, cytotoxic excitation light is necessary to obtain a fluorescence signal^14^. Additionally, two- (or multi-) photon microscopic analysis is necessary for deep tissue imaging. A bioluminescence probe using a luciferase reporter is another imaging probe which can be used to approach this problem. Although a substrate administration is necessary to obtain a bioluminescence signal, the signal can be detected without any excitation light^14^. Moreover, a bioluminescence signal can be detected from target tissues with a high signal-to-noise ratio^15^. We used a luciferase reporter as an imaging probe, and generated a novel transgenic mouse strain using the BAC clone containing the entire mouse *Bdnf* gene^16^. Using *Bdnf*-*Luciferase* transgenic (*Bdnf*-*Luc* Tg) mice, we successfully detected and visualized the change in *Bdnf* expression by measuring the bioluminescence signal *in vitro* ^16^. Here we examined whether the changes in *Bdnf* expression could be detected in living animals by *in vivo* bioluminescence imaging. We were able to detect the changes in *Bdnf* levels in the brain region as well as peripheral tissues such as adipose tissue, although an analogue of luciferase substrate producing near-infrared bioluminescence was necessary for detecting the changes in *Bdnf* levels in the brain. Our present study shows that the *Bdnf*-*Luc* Tg mouse would be a valuable tool for understanding changes in *Bdnf* levels under pathophysiological as well as physiological conditions and the role of *Bdnf* expression in living animals.

## Results

### Expression of *Bdnf*-*Luc* gene in *Bdnf*-*Luc* Tg mouse brain

**Figure 1:**
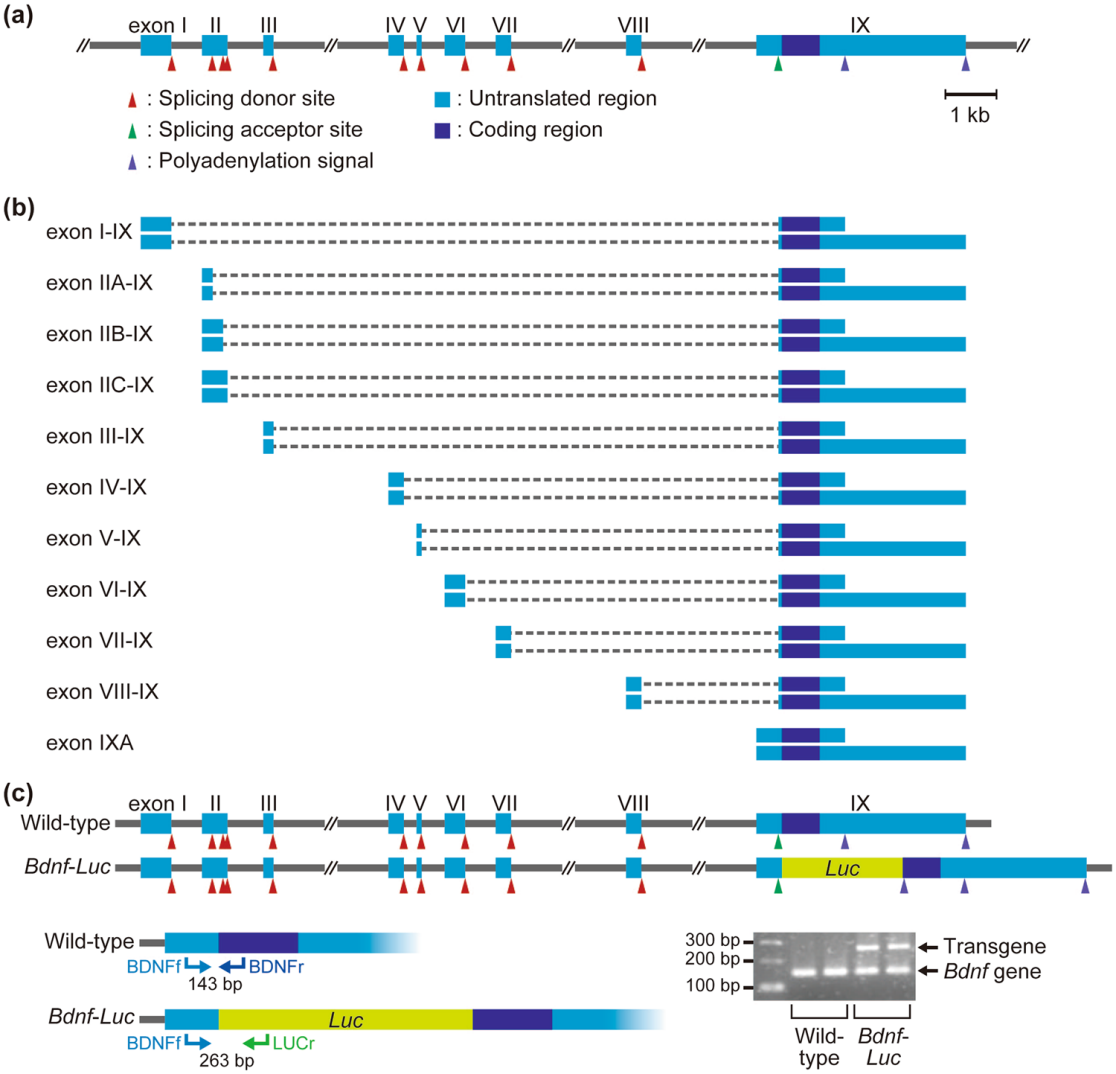
Generation of *Bdnf*-*Luc* Tg mice. (**a**,**b**) Structure of mouse *Bdnf* gene (**a**) and its multiple transcripts (**b**). Scale bar = 1 kb. (**c**) Schematic representation of wild-type *Bdnf* gene and luciferase-inserted transgene (*Bdnf*-*Luc*) in BAC and genotyping PCR result. Using three kinds of primers, BDNFf, BDNFr, and LUCr, genomic DNA extracted from mouse tail were amplified by PCR, and the PCR products were separated on 2% agarose gel. PCR products of *Bdnf* gene (143 bp) and *Bdnf*-*Luc* transgene (263 bp) are shown by arrows. A full-length gel is presented in Supplementary Figure 5.

**Figure 2:**
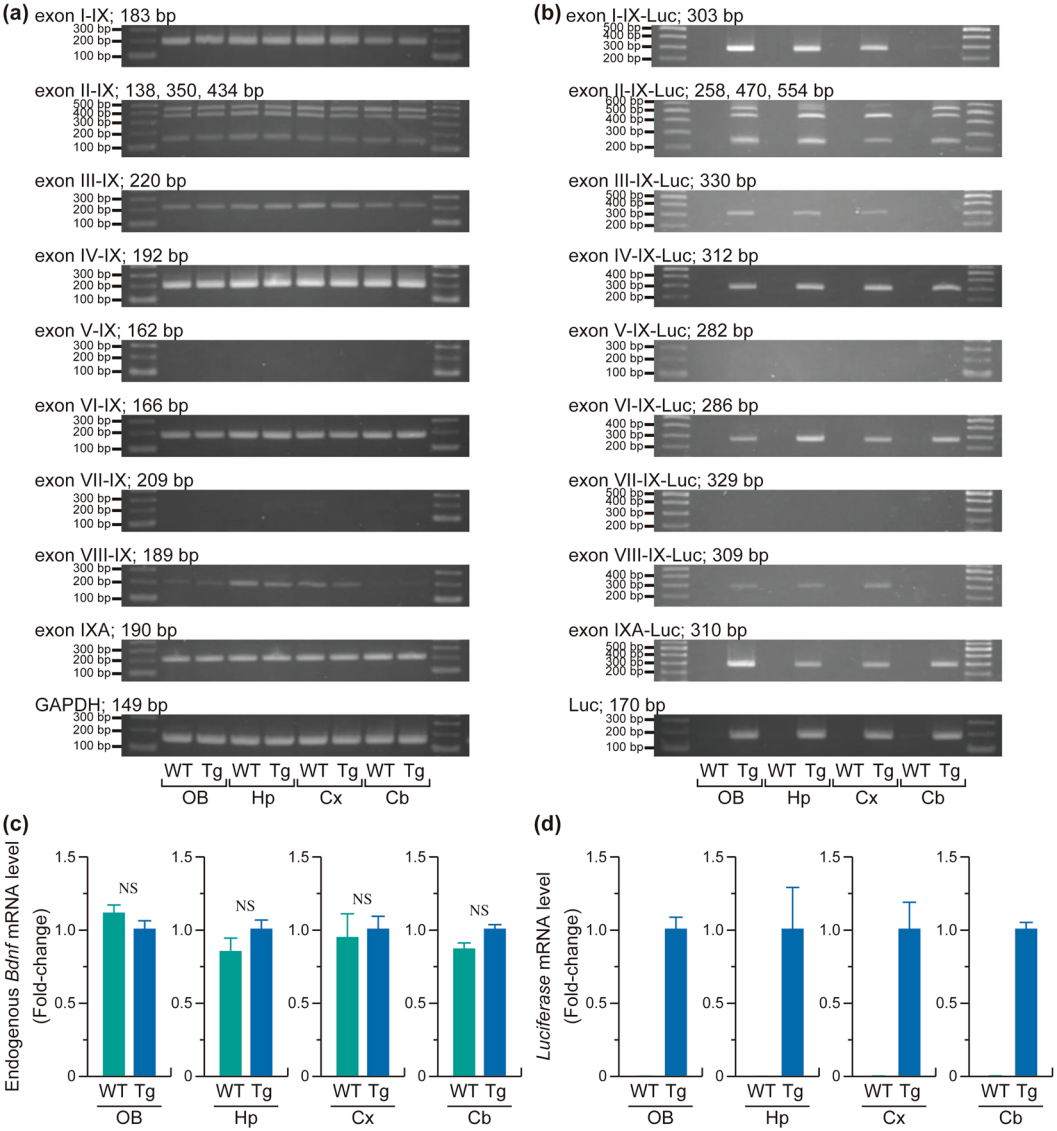
Expression of *Bdnf* and *Bdnf*-*Luc* mRNA in the brain of wild-type or *Bdnf*-*Luc* Tg mice. Total RNA was extracted from olfactory bulb (OB), hippocampus (Hp), cerebral cortex (Cx), and cerebellum (Cb) of wild-type (WT) or *Bdnf*-*Luc* Tg (Tg) mice. The expression of endogenous *Bdnf* (**a**) and *Bdnf*-*Luc* mRNA (**b**) was investigated by RT-PCR analysis. To distinguish alternative BDNF transcripts, we used a 5′ exon-specific primer and a common 3′ exon primer (refer to Methods). The experiment was performed independently at least three times, and the same tendency was observed. Full-length gels are presented in Supplementary Figure 5. The levels of endogenous *Bdnf* (**c**) and *Luc* (**d**) mRNA in WT or Tg mice were also measured by real-time PCR analysis. The expression level of each mRNA was normalized to that of *Gapdh* mRNA. The data represent the mean ± S.E.M. (n = 3). NS, not significant.

We previously generated *Bdnf*-*Luc* Tg mice by insertion of the *firefly luciferase* gene into the translation start site of the mouse *Bdnf* gene using a BAC clone containing the entire mouse *Bdnf* gene^16^ (Fig. 1). Mouse and rat *Bdnf* genes consist of nine exons. Multiple *Bdnf* transcripts were generated by a series of promoters, alternative splicing, and two distinct polyadenylation sites^17^ (Fig. 1). Using total RNA extracted from olfactory bulbs, hippocampus, cerebral cortex, and cerebellum of wild-type or *Bdnf*-*Luc* Tg littermates, we were able to detect an endogenous alternative *Bdnf* mRNA except exon V-IX and exon VII-IX, in both wild-type and the Tg brain (Fig. 2a). There was no significant difference between endogenous *Bdnf* mRNA in wild-type and the Tg mice (Fig. 2a,c and Supplementary Fig. 1). We could also detect *Luc* transcript and PCR products at the expected size of *Bdnf*-*Luc* mRNA in the Tg, but not wild-type, mouse brain (Fig. 2b,d), indicating that the expression of *Bdnf*-*Luc* mRNA from the *Bdnf*-*Luc* transgene occurs normally.

### *In vivo* bioluminescence imaging of *Bdnf*-*Luc* Tg mice

**Figure 3:**
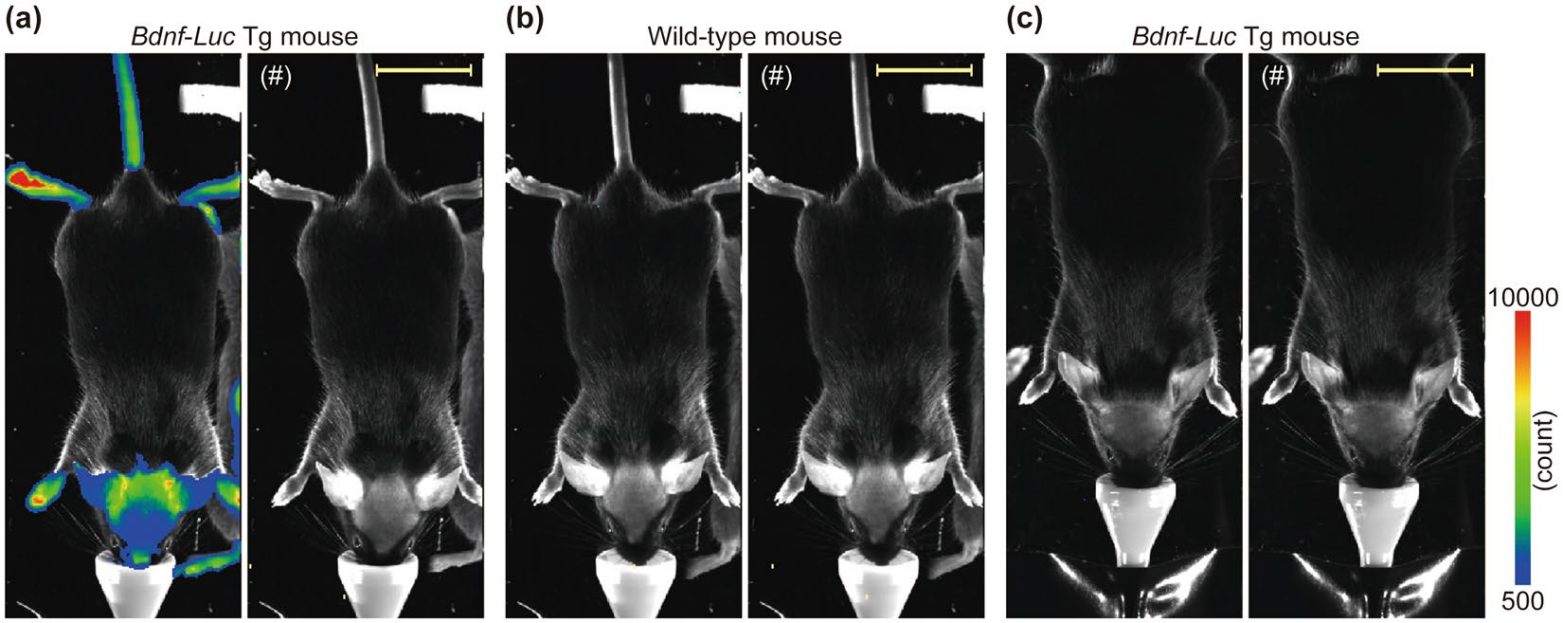
*In vivo* bioluminescence imaging using *Bdnf*-*Luc* Tg mice. The fur on the head of the mouse was shaved before *in vivo* bioluminescence imaging. The imaging was performed using *Bdnf*-*Luc* Tg (**a**,**c**) and wild-type (**b**) mice. Ten minutes after intraperitoneal administration of d-luciferin (150 mg/kg body weight, **a**,**b**) or saline (**c**) under anesthesia, the bioluminescence was measured by *in vivo* imaging (Exposure: 2 min). The intensity of the bioluminescence signal is shown in pseudocolored images. The photograph of the Tg or wild-type mouse (indicated by (#)) was merged with the pseudocolored image. Scale bar = 20 mm. The experiment was performed independently at least four times, and the same tendency was observed.

We previously showed visualization of *Bdnf* expression in primary cultures of cortical cells prepared from *Bdnf*-*Luc* Tg mouse embryos^16^ (Supplementary Fig. 2). We here investigated whether the bioluminescence signal could be detected in *Bdnf*-*Luc* Tg mice. After shaving the fur from the head of the Tg mice, a luciferase substrate, d-luciferin, was administered to the Tg mice. After the administration of d-luciferin, we successfully detected the bioluminescence signal from the head region of the Tg mice (Fig. 3a). In contrast, the signal was never observed in wild-type mice (Fig. 3b). Additionally, no bioluminescence signal was observed when saline was administered to the Tg mice (Fig. 3c). After administration of d-luciferin in Tg mice, the bioluminescence signal was also detected in ears, limbs, and tail, in addition to the head region (Fig. 3a), suggesting that the signal was detected from surface tissues such as skins. Therefore, we removed the head skin of the Tg mice before *in vivo* imaging to examine the changes in the signal in the brain region. Actually, using the head skin removed from the Tg mice before *in vivo* imaging, we could detect the expression of endogenous *Bdnf* mRNA while the levels of the mRNA in the skin tissue were quite lower than those in the cerebral cortex (Supplementary Fig. 3a,b). In addition, the bioluminescence signal was also observed from the isolated head skin (Supplementary Fig. 3c). Furthermore, we found the expression of *Luc* mRNA in the skin tissues prepared from the Tg but not wild-type mice (Supplementary Fig. 3d). These results indicate that the bioluminescence signals, which were detected from ears, limbs, and tail (probable skin regions), are due to *Bdnf* expression rather than artificial effects.

### *In vivo* imaging using an analogue of d-luciferin, TokeOni, producing near-infrared bioluminescence

**Figure 4:**
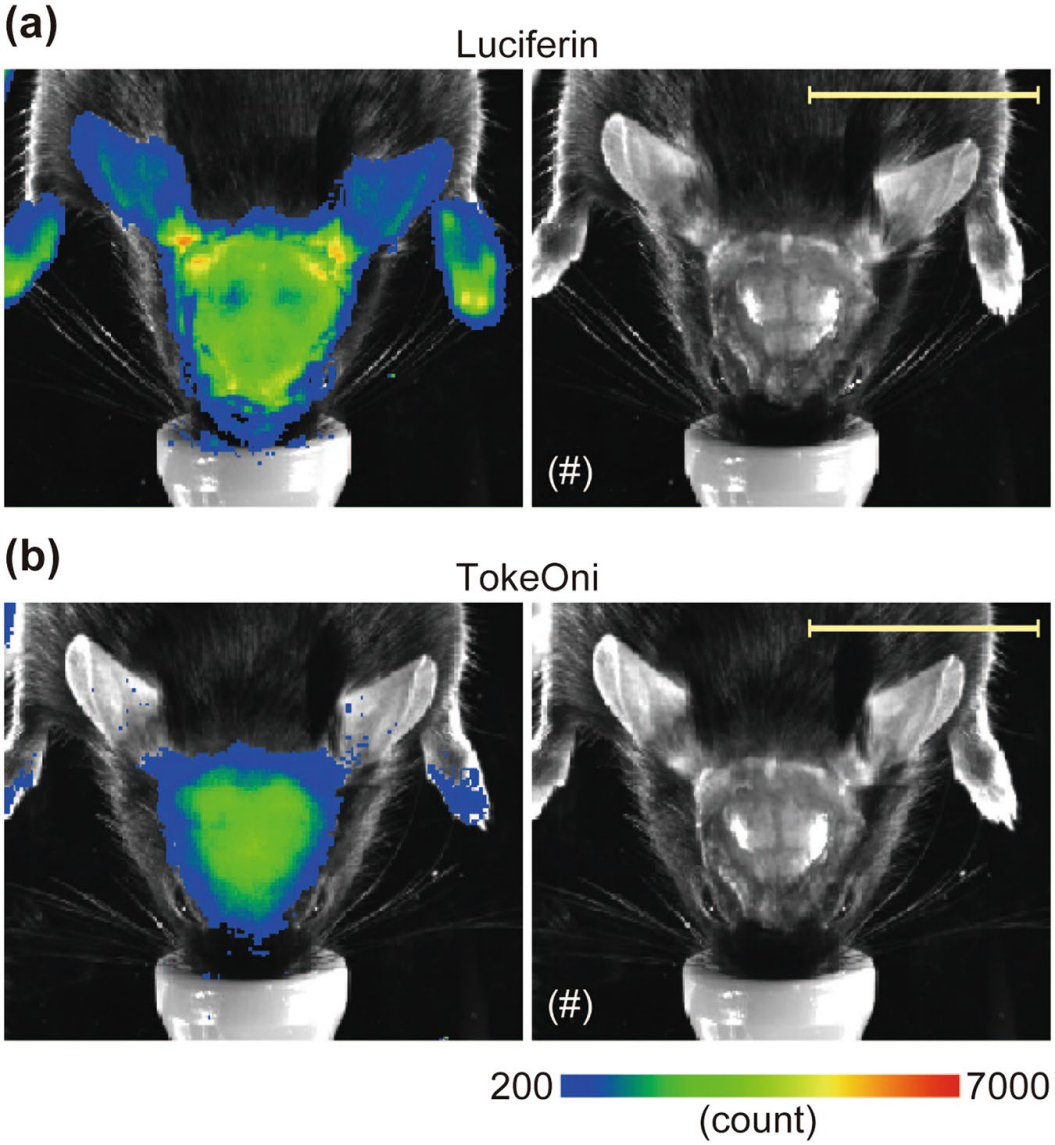
Comparison of the bioluminescence signal obtained by d-luciferin and that by an analogue of d-luciferin, TokeOni. The bioluminescence was first measured using d-luciferin (**a**). Three hours after the measurement, the bioluminescence was re-measured using TokeOni (**b**). These substrates were administered to the Tg mice intraperitoneally at the same dose (150 mg/kg body weight) and under the same conditions (Exposure: 2 min). The photograph of the head region of the Tg mouse (indicated by (#)) was merged with the pseudocolored image. Scale bar = 20 mm. The experiment was performed independently at least three times, and the same tendency was observed.

Because the emission maximum of bioluminescence generated by d-luciferin is 562 nm, the light has poor penetration across biological tissues. Accordingly, it is hard to detect the bioluminescence signal derived from deep tissues when d-luciferin is used as a substrate of luciferase. Although we could detect the bioluminescence signal in *Bdnf*-*Luc* Tg mice after d-luciferin administration, it was difficult to identify which signal was derived from the brain even if the skin of the head region was removed (Fig. 4a). Recently, it was reported that TokeOni, which is an analogue of d-luciferin that generates near-infrared bioluminescence, allows for the detection of the signal derived from deep tissues^18^. We could clearly detect the bioluminescence signal from the brain region of Tg mice when TokeOni was administered intraperitoneally (Fig. 4b). Furthermore, the signal from probable skin regions such as ears was decreased (Fig. 4b). Thus, it is suggested that TokeOni is a more appropriate substrate for bioluminescence imaging of the brain particularly in *Bdnf*-*Luc* Tg mice.

### Detection of increases in *Bdnf* expression in response to PACAP by *in vivo* imaging

**Figure 5:**
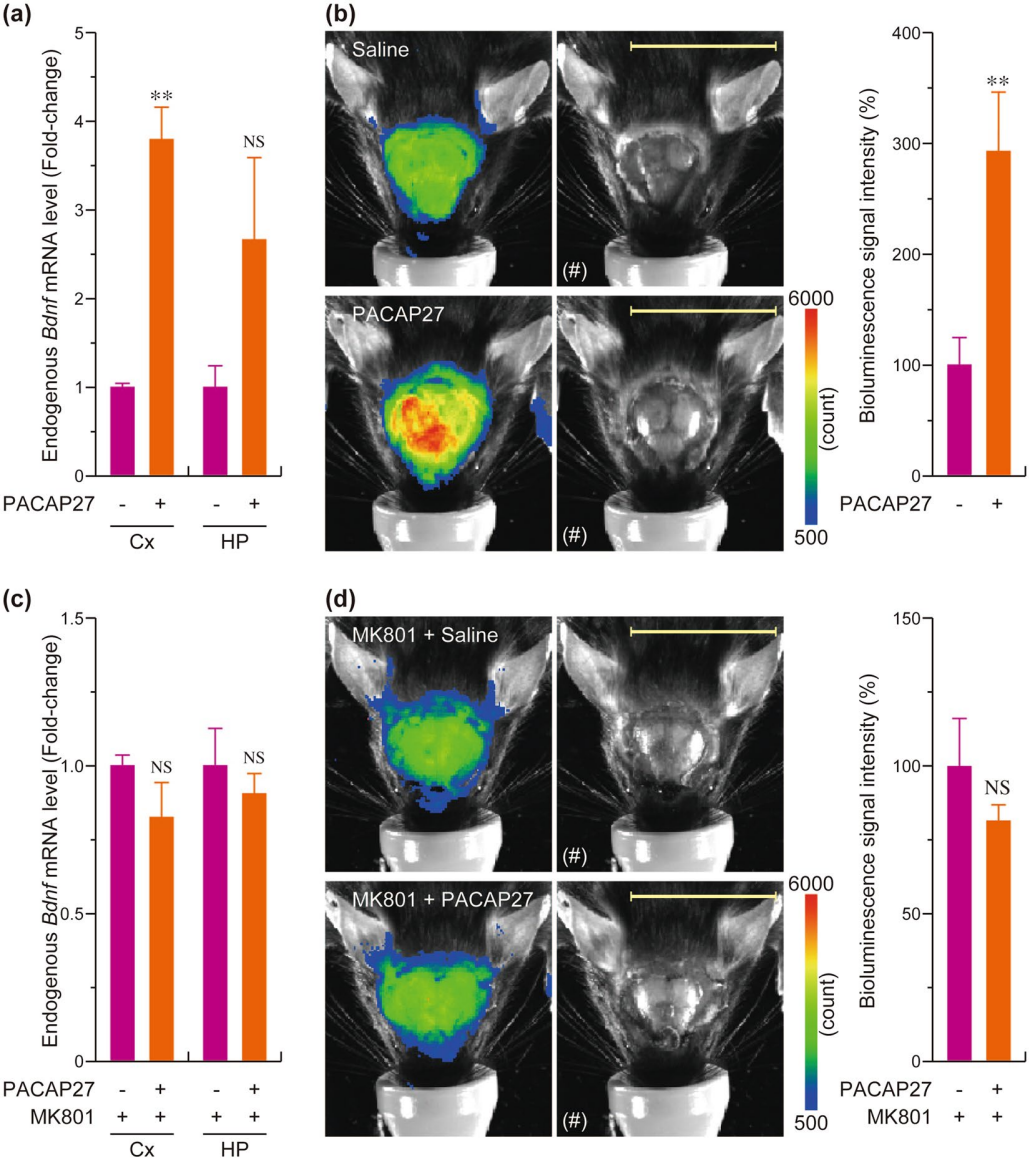
Detection of the increase in *Bdnf* expression in cerebral cortex after intracerebroventricular injection of PACAP27 by *in vivo* bioluminescence imaging. (**a**) *Bdnf*-*Luc* Tg mice were intracerebroventricularly administered saline or 0.1 mM PACAP27 (Injection volume: 5 μl. Injection site: Bregma ± 0 mm, 1 mm from midline, 3 mm depth from the skull surface). Three hours after the injection, total RNA was extracted from cerebral cortex and hippocampus of the Tg mice. The expression of endogenous *Bdnf* mRNA was investigated by RT-PCR analysis. The data represent the mean ± S.E.M. (n = 4–5). **p < 0.01 versus saline-injected mice. NS, not significant. (**b**) Representative images of the bioluminescence signal of *Bdnf*-*Luc* Tg mice and changes in the bioluminescence signal intensity in saline- or PACAP27-injected Tg mice. Six hours after the injection, TokeOni (75 mg/kg body weight) was administered intraperitoneally to the Tg mice, and the bioluminescence was measured by *in vivo* imaging (Exposure: 3 min). The data represent the mean ± S.E.M. (n = 6). The photograph of the head region (indicated by (#)) was merged with the pseudocolored image. Scale bar = 20 mm. (**c**,**d**) The same experiments shown in Fig. 5a and b were performed using the Tg mice pretreated with MK801. MK801 (1 mg/kg body weight) was intraperitoneally injected to the mice 30 min before the saline- or PACAP-injection. The data represent the mean ± S.E.M. (n = 3). NS, not significant.

We previously reported that intracerebroventricular injection of pituitary adenylate cyclase-activating polypeptide (PACAP), which is a member of the vasoactive intestinal polypeptide (VIP)/secretin/glucagon family and participates in a variety of neural functions^19^, significantly increased the levels of *Bdnf* mRNA in the cerebral cortex of mice^16^. We also demonstrated that the levels of *Bdnf* mRNA significantly increased in the cortex after the intracerebroventricular injection of PACAP into the Tg mice, and the increase tended to be observed in the hippocampus (Fig. 5a). Therefore, we next examined whether this increase in *Bdnf* mRNA in the brain region could be detected by *in vivo* bioluminescence imaging with TokeOni. We measured the bioluminescence signal 6 h after the intracerebroventricular injection of saline or PACAP. We found that the bioluminescence signal of the brain region in the PACAP-injected Tg mice was higher than that in the saline-injected mice (Fig. 5b). Quantification of the signal also indicated significant increases in the signal associated with PACAP injection (Fig. 5b). Furthermore, consistent with our previous report, the PACAP-induced increase in *Bdnf* expression and the bioluminescence signals was not observed when the Tg mice were pretreated with an antagonist of *N*-methyl-d-aspartate receptor (NMDAR), MK801 (Fig. 5c,d). Therefore, the changes in *Bdnf* expression in the brain regions can be clearly visualized by *in vivo* bioluminescence imaging with a luciferin analogue generating near-infrared bioluminescence.

### Strong correlation between increases in body weight and bioluminescence signal in the abdominal region of *Bdnf*-*Luc* Tg mice under a high-fat diet

**Figure 6:**
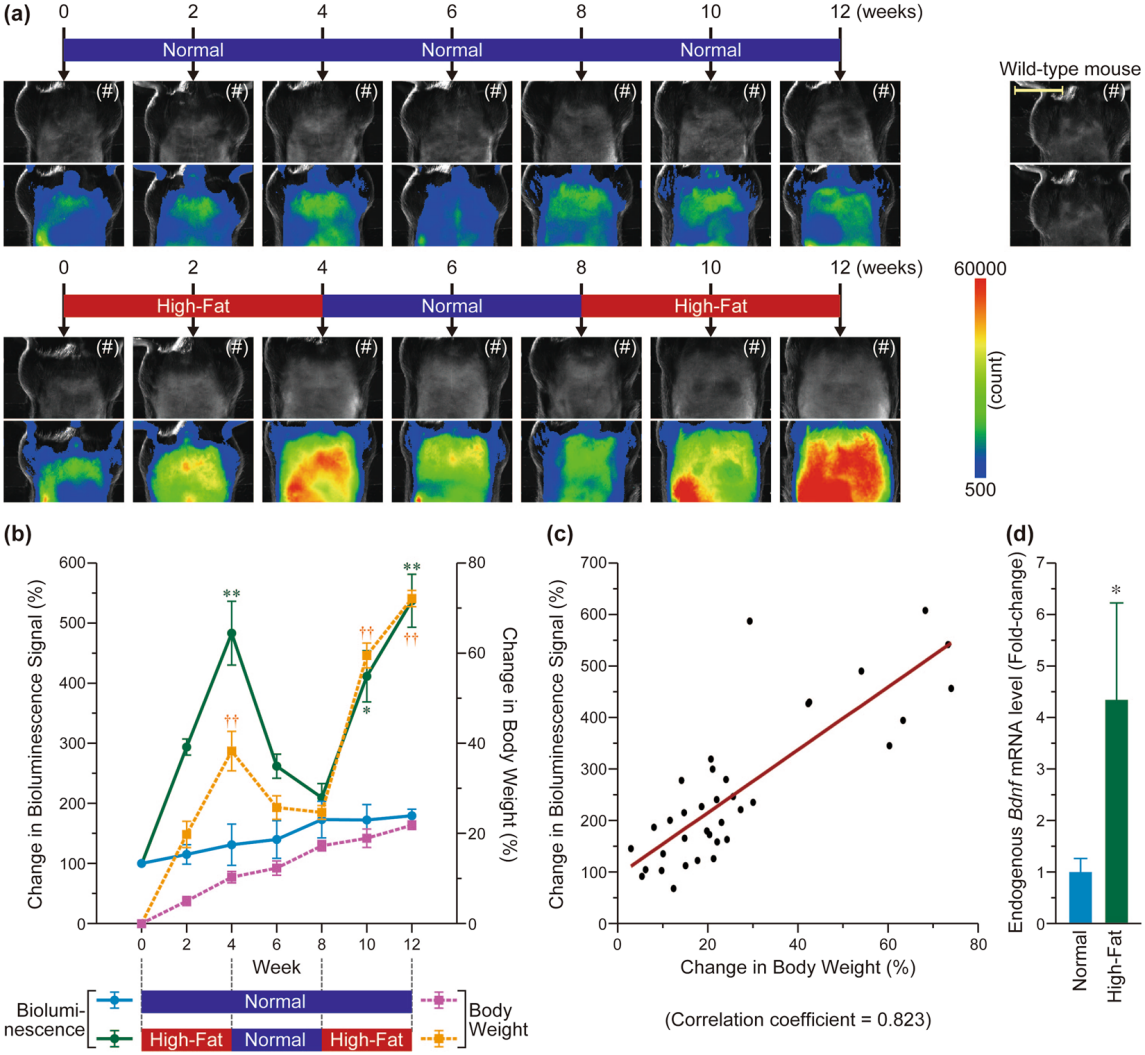
Strong correlation between the increase in body weight and bioluminescence signal under normal or high-fat diet in *Bdnf*-*Luc* Tg mice. (**a**) Representative images of the bioluminescence signal of *Bdnf*-*Luc* Tg mice under normal or high-fat diet. Imaging was performed with d-luciferin because it was sufficient to measure the changes in the signal from the abdominal region, which probably emanated from subcutaneous adipose tissues. In the normal diet group, we fed the normal diet to Tg mice continuously. In the high-fat diet group, we fed the high-fat diet to the mice for 4 weeks, and then the high-fat diet was changed to the normal diet. Four weeks after the change, we fed the high-fat diet to the Tg mice again. We also confirmed that the bioluminescence signal was not detected in the abdominal region of wild-type mice. Scale bar = 20 mm. The photograph of the abdominal region (indicated by (#)) was merged with the pseudocolored image. (**b**) Changes in the bioluminescence signals (circle) and body weight (square) of normal diet (light blue or pink) or high-fat diet (green or orange)-fed *Bdnf*-*Luc* Tg mice. The data represent the mean ± S.E.M. (n = 3). *p < 0.05, **p < 0.01, and ^††^ *p* < 0.01 versus normal diet-fed Tg mice. (**c**) The results shown in (**b**) were plotted, and statistical analysis was performed using Pearson’s correlation coefficient test (*rs* = 0.823, *p* < 0.001). (**d**) After the last measurement of the bioluminescence signals (12 weeks), subcutaneous adipose tissues of each Tg mouse were isolated and total RNA was extracted by TRIsure. Then, the expression levels of endogenous *Bdnf* mRNA was examined by real-time PCR analysis. The data represent the mean ± S.E.M. (n = 9). *p < 0.05 versus normal diet-fed mice.

Although d-luciferin was unlikely to be suitable for *in vivo* imaging of brain regions, we always detected the signal from skin, limbs, tail, and the abdominal region in *Bdnf*-*Luc* Tg mice by *in vivo* bioluminescence imaging with d-luciferin. We sometimes found that some aged mice (approximately 1-year-old) exhibited high body weight and others did not. During *in vivo* imaging analysis with these aged Tg mice, we incidentally observed that the bioluminescence signal of the abdominal region in the aged Tg mice with higher body weight was higher than that for mice with lower body weight (Supplementary Fig. 4a). Consistently, the levels of endogenous *Bdnf* mRNA in the adipose tissues of the Tg mice with higher body weight were also higher (Supplementary Fig. 4b). This suggested that the expression of *Bdnf* in adipose tissues may correlate with metabolic abnormalities such as obesity. To address this issue, we here fed *Bdnf*-*Luc* Tg mice a normal or high-fat diet, and the body weight and bioluminescence signal of the abdominal region of the Tg mice were measured every 2 weeks. The body weight of normal diet-fed Tg mice gradually increased and the bioluminescence signal of the abdominal region hardly increased (Fig. 6a,b). In contrast, the high-fat diet was associated with increasing body weight (adipose tissues clearly increased in these mice), which was accompanied by an increased abdominal bioluminescence signal (Fig. 6a,b). Interestingly, the increased body weight and bioluminescence signal were reduced when the high-fat diet was exchanged with a normal one (Fig. 6a,b). Furthermore, subsequent administration of the high-fat diet significantly re-increased both (Fig. 6a,b). Statistical analysis revealed a strong correlation between the increase in body weight and bioluminescence signal intensity (Fig. 6c). After the final *in vivo* imaging, total RNA was extracted from subcutaneous adipose tissue, and the levels of *Bdnf* mRNA were examined by RT-PCR. We found that the levels of endogenous *Bdnf* mRNA in adipose tissue isolated from high-fat diet-fed Tg mice were higher than that isolated from normal diet-fed mice (Fig. 6d). In further experiments, we fed *Bdnf*-*Luc* Tg mice normal or high-fat diets for 2 weeks, and then measured the bioluminescence signal of the abdominal region (Supplementary Fig. 5a). After detecting an increased abdominal bioluminescence signal, total RNA and total protein were extracted from subcutaneous adipose tissue, and levels of *Bdnf* mRNA and BDNF protein were examined by RT-PCR and immunoblotting, respectively. Consistent with the increased bioluminescence signal, the high-fat diet significantly increased *Bdnf* mRNA levels (Supplementary Fig. 5b). A slight increase in BDNF protein levels was also observed in adipose tissue isolated from high-fat diet-fed Tg mice (Supplementary Fig. 5c). Therefore, the high-fat diet was associated with increases in the levels of *Bdnf* expression (*Bdnf* mRNA in particular) in adipose tissue, accompanied by increases in body weight.

## Discussion

We previously generated a novel transgenic mouse strain, *Bdnf*-*Luc* Tg mice, and successfully visualized the changes in *Bdnf* expression in living cells. In this study, we showed that the changes in expression could also be monitored by *in vivo* bioluminescence imaging with *Bdnf*-*Luc* Tg mice. This is the first report showing a method for monitoring changes in *Bdnf* expression in living animals.

The bioluminescence signal was observed not only in the head region but also in other areas such as ears, limbs, and tail. BDNF is expressed in non-neuronal cells such as hair follicles and keratinocytes^20–22^; therefore, it is plausible that the bioluminescence signal was detected in skin regions of the Tg mice. In support of these findings, we here found that, (1) endogenous *Bdnf* mRNA was expressed in isolated head skin, (2) the bioluminescence signals were detected from the skin tissue of the Tg mice, and (3) the expression of *Luc* mRNA was observed in the skin removed from the Tg but not wild-type mice. Because the bioluminescence signal was detected in the head region of the Tg mice after removing skin from the head region, this signal should be derived from the brain region. However, the bioluminescence signal obtained by d-luciferin is a poorly tissue-penetrating light because of its short-wavelength (562 nm), and it was hard to identify which signals were derived from the brain tissue.

Detection of the bioluminescence signal in the brain region of the *Bdnf*-*Luc* Tg mice was nicely improved by the use of TokeOni, an analogue of d-luciferin. As described above, we detected the bioluminescence signal from probable skin region of the Tg mice when d-luciferin was used. Although the expression of endogenous *Bdnf* in the skin is lower than that in the brain, detection of the bioluminescence signal from the skin is more likely because the skin is a body surface tissue (it is easy to detect the bioluminescence from a body surface tissue because of the absence of interfering tissue). However, the brightness of the total bioluminescence generated by TokeOni is lower than that produced by d-luciferin^18^. This is one of the reasons why the signal from skin regions (ear, limbs, and others) was reduced when TokeOni was used. Instead, the signal from the brain region was clearly detected with TokeOni in spite of its lower brightness. It has been also reported that the bioluminescence signal obtained by TokeOni was maximal at lower concentrations compared with d-luciferin^18^. These properties of TokeOni could be reasons for improvements in detection of the signal from the brain regions. Furthermore, we successfully observed the increased *Bdnf* expression and bioluminescence signal in the cerebral cortex of the Tg mice after intracerebroventricular administration of PACAP. Consistent with our previous findings that this PACAP-induced *Bdnf* expression was dependent on NMDAR^16^, intraperitoneal injection of MK801 completely blocked this inducible effect of PACAP on *Bdnf* expression. These results indicate that *Bdnf*-*Luc* Tg mice with TokeOni would be a valuable tool for monitoring the changes in *Bdnf* expression in the brain region of the living mice. And also, these data strongly suggest that TokeOni as well as d-luciferin can be transported across the brain-blood barrier.

The bioluminescence signal was likely to be easily detected particularly in peripheral regions neighboring the body surface, even if d-luciferin was used. In this study, we detected higher signal from abdominal regions of aged Tg mice with high body weight. Based on this observation, we fed the Tg mice a normal or high-fat diet, and found that a high-fat diet significantly increased the bioluminescence signal in abdominal regions, probably in adipose tissue, and showed a strong correlation between the increase in the bioluminescence signal and body weight in the high-fat diet-fed Tg mice. In fact, we observed the higher expression levels of endogenous *Bdnf* mRNA in the adipose tissues isolated from the high-fat diet-fed Tg mice. Consistent with our present study, it has been recently reported that *Bdnf* is expressed in adipose tissue, and the expression is significantly increased by high-fat diet feeding^23^. In pathological adipose tissue expansion, adipose tissues are poorly oxygenated because adipocyte hypertrophy generates hypoxia^24^. This hypoxic condition induces a master regulator of oxygen homeostasis, hypoxia-inducible factor (HIF) 1α, and M1-type macrophages prevail, leading to chronic inflammation in adipose tissues^24^. Previous reports indicate that hypoxia induces BDNF expression in some cells, such as adipose-derived mesenchymal stem cells^25^ and human pulmonary artery smooth muscle cells^26^. Although it has been reported that high-fat diet feeding significantly increased *Bdnf* expression in adipose tissues, as described above, the results obtained by the adipocyte-specific *Bdnf* knockout strategy revealed that *Bdnf* is not expressed in adipocytes^23^. Considering the previous report showing that BDNF is expressed in M1 macrophages^27^, it is possible that a high-fat diet-induced pathological adipose tissue expansion creates hypoxia, which induces HIF1α levels and prevailing M1 macrophages, resulting in chronic inflammation and BDNF induction. In addition, because BDNF stimulates HIF1α expression^28, 29^, BDNF induction in pathological adipose tissue expansion might exacerbate chronic inflammation in the adipose tissues. Further study is necessary to uncover the role of BDNF in pathological obesity. Reversible changes in the bioluminescence signal were observed in the Tg mice under the high-fat and normal diet conditions; therefore, the Tg mice could be a potent tool for examination of the role of *Bdnf* expression in metabolic homeostasis as well as obesity-related pathophysiology. However, although increased bioluminescence signal from the abdominal region strongly correlated with higher levels of endogenous *Bdnf* mRNA in adipose tissue, endogenous BDNF protein levels only slightly increased in the same tissue. Luciferase protein produced in the *Bdnf*-*Luc* Tg mice is not a secretory protein, whereas BDNF is a secretory factor, which may explain why *Bdnf* mRNA levels do not correlate well with BDNF protein in adipose tissue isolated from high-fat diet-fed Tg mice. Alternatively, mismatch between *Bdnf* mRNA and BDNF protein has also been reported in the developing visual cortex: before eye opening, many neurons in the visual cortex express *Bdnf* mRNA but not BDNF protein^30^, suggesting that *Bdnf* mRNA levels do not always correspond with BDNF protein. In any case, even though it might be hard to detect changes in BDNF protein expression using *Bdnf*-*Luc* Tg mice, our present data strongly support that changes in *Bdnf* mRNA levels can be monitored using Tg mice.

Taken together, our results show a detection of the change in *Bdnf* expression using *Bdnf*-*Luc* Tg mice. Because the change in *Bdnf* levels can be monitored in living mice, we can continuously investigate the levels of *Bdnf* expression while measuring physiological and behavioral alterations. Thus, the Tg mouse would be a powerful tool for investigating alterations in *Bdnf* levels in pathophysiological as well as physiological conditions, and could help us better understand the role of *Bdnf* expression in physiological functions and diseases.

## Methods

### Animals

All animal care and experiments were approved by the Animal Experiment Committee of the University of Toyama (Authorization No. S-2010 MED-51, A2011PHA-18, and A2014PHA-1), and were carried out in accordance with the Guidelines for the Care and Use of Laboratory Animals of the University of Toyama. Mice were housed under standard laboratory conditions (12 h–12 h/light–dark cycle with light on at 7:00 am, room temperature at 22 ± 2 °C) and had free access to food and water. Generation of *Bdnf-Luc* Tg mice was described previously^16^ (Fig. 1). Wild-type littermates were used as control animals (Figs 2, 3 and 6a, Supplementary Figs 1 and 3d). In this study, 8–12 week-old male and female mice were used.

### Reagents

Endotoxin-free d-luciferin (d-Luciferin EF) was purchased from Promega (Madison, WI, USA). A d-luciferin analogue, TokeOni, was kindly donated from Kuroganekasei Co. Ltd. PACAP27 was purchased from the Peptide Institute, Inc (Ibaraki-shi, Osaka, Japan).

### RT-PCR analysis

Total RNA was extracted from wild-type and *Bdnf*-*Luc* Tg mice using TRIsure (BIOLINE, London, UK), and quantified with a NanoDrop spectrophotometer. One microgram of RNA was reverse-transcribed into cDNA in 10 μL of a reaction mixture containing 500 nM oligo(dT)15 (5′-AAGCTTTTTTTTTTV-3′) as a primer, 100 units of SuperScript II reverse transcriptase (Invitrogen, Carlsbad, CA, USA), 0.5 mM of each dNTP (Invitrogen), 1 × first-strand buffer, 10 mM DTT, and 5 units of RNase inhibitor (Invitrogen). The reaction was performed at 42 °C for 50 min and stopped by incubation at 70 °C for 15 min. The resulting first-strand cDNA solution was diluted four-fold with water (diluted cDNA solution). PCR was performed using GoTaq Flexi DNA polymerase (Promega), according to the manufacturer’s instructions. The PCR product was separated on a 2% agarose gel. The primer sequences to detect spliced *Bdnf* mRNA were as follows: exon I-S; 5′-CAAACAAGACACATTACCTTCCTGC-3′, exon II-S; 5′-AGCCAGCGGATTTGTCCGA-3′, exon III-S; 5′-CTCCCCGAGAGTTCCG-3′, exon IV-S; 5′-GGAAATATATAGTAAGAGTCTAGAACCTTGG-3′, exon V-S; 5′-CTCTGTGTAGTTTCATTGTGTGTTCG-3′, exon VI-S; 5′-GGACCAGAAGCGTGACAAC-3′, exon VII-S; 5′-AAAGGGTCTGCGGAACTCCA-3′, exon VIII-S; 5′-GACTGTGCATCCCAGGAGAA-3′, exon IXA-S; 5′-GGTCTGAAATTACAAGCAGATGGG-3′, exon IXA-AS; 5′ GACGTTTACTTCTTTCATGGGCG-3′, Luc-AS; 5′-CGTTGTAGATGTCGTTAGCTGG-3′. 5′ exon-specific primer and exon IXA-AS, or 5′ exon-specific primer and Luc-AS, were used to discriminate spliced *Bdnf* (Fig. 2a) and *Bdnf*-*Luc* (Fig. 2b and Supplementary Fig. 1) mRNA, respectively. The primer sequences to detect *Gapdh* and *Luc* mRNA were as follows: *Gapdh*; 5′-GCACAGTCAAGGCCGAGAA-3′ and 5′-CTTCTCCATGGTGGTGAAGAC-3′, *Luciferase*; 5′-CGAGTACTTCGAGATGAGCG-3′ and 5′-CGTTGTAGATGTCGTTAGCTGG-3′. Realtime PCR amplification was performed using the Stratagene Mx3000p Real-Time PCR system in a 20 μL reaction mixture containing 1 × Brilliant SYBR Green QPCR Master Mix (Stratagene, La Jolla, CA, USA), 2 μL of diluted cDNA solution, 200 nM primer pairs, and 30 nM ROX reference dye. The thermal profile for PCR included initial denaturation at 95 °C for 10 min, followed by 45 cycles of denaturation at 95 °C for 45 s, annealing at 57 °C for 45 s, and extension at 72 °C for 1 min. The primer sequences were as follows: total *Bdnf*; 5′-AAGGACGCGGACTTGTACAC-3′ and 5′-CGCTAATACTGTCACACACGC-3′, *Luc* and *Gapdh* are shown above.

### *In vivo* imaging

In Fig. 3, after the fur on the head of *Bdnf*-*Luc* Tg mice was shaved under anesthesia, *in vivo* bioluminescence imaging was performed. In Figs 4 and 5, one week before *in vivo* imaging, the head skin of *Bdnf*-*Luc* Tg mice was removed under anesthesia. The Tg mice were anesthetized by inhalation of 1.5% isoflurane before and during imaging. d-luciferin EF (Promega), dissolved in saline, was administered intraperitoneally (150 mg/kg body weight) to the Tg mice. TokeOni was also used as a luciferase substrate generating near-infrared bioluminescence. TokeOni, dissolved in water, was administered intraperitoneally (150 mg/kg body weight (Fig. 4) or 75 mg/kg body weight (Fig. 5)) to the Tg mice. After the substrate injection, the bioluminescence signal intensity in the Tg mice was measured using an *in vivo* imaging system (Clairvivo OPT; Shimadzu Co., Kyoto, Japan) consisting of a dark chamber and a cooled charge-coupled device (CCD) camera (PIXIS-2048B, Roper Industries, Sarasota, FL, USA). Bioluminescence images were taken for 2 min (Figs 3, 4 and 6) or 3 min (Fig. 5) with 4 × 4 binning without the use of an optical filter. Pseudocolored luminescent images representing the spatial distribution of emitted photons were overlaid on photographs of mice taken in the chamber under a dim light.

### *In vivo* imaging of mice given normal or high-fat diet

Before *in vivo* imaging, the fur of the abdominal region of the Tg mice was shaved because the black fur attenuates photon emission. After measuring bioluminescence signals with d-luciferin, *Bdnf*-*Luc* Tg mice were given a normal diet (CREA Japan, Inc., Tokyo, Japan) or a high-fat diet (D12451, Research Diets Inc., New Brunswick, NJ, USA). The bioluminescence signal and body weight of the Tg mice were measured every two weeks.

### Immunoblotting

Total protein from adipose tissue was prepared using the Total Protein Extraction Kit for Adipose Tissues and Cultured Adipocytes (101 Bio, LLC, Palo Alto, CA, USA), according to the manufacturer’s instruction. Protein extracts (10 μg) were separated by SDS-PAGE on 15% (BDNF) or 10% (α-tubulin) gels. Proteins were transferred to PVDF membranes, and the membranes were incubated with anti-BDNF antibody (1:200; Alomone Labs Ltd., Jerusalem, Israel) or anti-α-tubulin antibody (1:1000; Sigma-Aldrich, St. Louis, MO, USA) overnight at 4 °C. Next, membranes were incubated with HRP-conjugated rabbit or mouse anti-IgG (1:5000; GE Healthcare, Buckinghamshire, England) for 1 h at RT. Protein levels were detected using ECL Western blotting detection reagents (GE Healthcare).

### Statistical analysis

All data are presented as the mean ± the standard error of the mean (S.E.M.). Statistical analyses were performed using the Student’s *t* test followed by the F-test (Fig. 5), one-way ANOVA with Scheffé’s *F*-test (Fig. 6b), Pearson’s correlation coefficient test (Fig. 6c), and two-sided Mann-Whitney’s *U*-test (Fig. 6d).

## Electronic supplementary material

Supplementary information
