# Supplementary material for: Visualizing changes in brain-derived neurotrophic factor (BDNF) expression using bioluminescence imaging in living mice
Source: Sci Rep. 2017 Jul 10;7:4949. doi: 10.1038/s41598-017-05297-x (PMC5504055; doi:10.1038/s41598-017-05297-x)

Supplementary information

Visualizing changes in brain-derived neurotrophic factor (BDNF) expression using bioluminescence imaging in living mice

Mamoru Fukuchi[1,4,*], Hironori Izumi[2], Hisashi Mori[2], Masahiro Kiyama[3], Satoshi Otsuka[3], Shojiro Maki[3], Yosuke Maehata[4], Akiko Tabuchi[4], and Masaaki Tsuda[4]

[1]Laboratory of Molecular Neuroscience, Faculty of Pharmacy, Takasaki University of Health and Welfare, 60 Nakaorui-machi, Takasaki-shi, Gunma 370-0033, Japan

[2]Department of Molecular Neuroscience, Graduate School of Medicine and Pharmaceutical Sciences, University of Toyama, 2630 Sugitani, Toyama-shi, Toyama 930-0194, Japan

[3]Department of Engineering Science, Graduate School of Informatics and Engineering, The University of Electro-Communications, 1-5-1 Chofugaoka, Chofu-shi, Tokyo 182-8585, Japan

[4]Department of Biological Chemistry, Graduate School of Medicine and Pharmaceutical Sciences, University of Toyama, 2630 Sugitani, Toyama-shi, Toyama 930-0194, Japan

*Corresponding author: Mamoru Fukuchi

Laboratory of Molecular Neuroscience, Faculty of Pharmacy, Takasaki University of Health and Welfare, 60 Nakaorui-machi, Takasaki-shi, Gunma 370-0033, Japan

Tel: +81-27-352-1180 (ext. 8317), Fax: +81-27-352-1118, e-mail: fukuchi@takasaki-u.ac.jp

Supplementary figure legends

Supplementary Figure 1. Expression of endogenous *Bdnf* mRNA in the brain of wild-type or *Bdnf-Luc* Tg mice.

Total RNA was extracted from olfactory bulb (OB), hippocampus (Hp), cerebral cortex (Cx), and cerebellum (Cb) of wild-type (WT) or *Bdnf-Luc* Tg (Tg) mice. The expression levels of endogenous *Bdnf* mRNA were measured by real-time PCR analysis. To distinguish alternative BDNF transcripts, we used a 5' exon-specific primer and a common 3' exon primer (refer to Methods). The expression level of each mRNA was normalized to that of *Gapdh* mRNA. The data represent the mean ± S.E.M. (n = 3). NS, not significant. Among multiple *Bdnf* transcripts, the expression of *Bdnf* exon V-IX and VII-IX mRNA was not detected, consistent with the results obtained by semi-quantitative RT-PCR analysis (Fig. 2). The levels of *Bdnf* exon III- IX and VIII-IX mRNA were lower, therefore, these fold-change values varied among samples.

Supplementary Figure 2. Visualization of depolarization-induced BDNF induction *in vitro*.

(a) At 5 days in culture, primary cultures of cortical cells prepared from *Bdnf-Luc* mice embryos (E16.5) were treated with 25 mM KCl for 6 h, and cell lysate was prepared for measuring luciferase activity. Luciferase activity was measured by a Luciferase assay kit (Promega). Nicardipine (5 μM) was added 10 min before the KCl treatment. Data represent the mean ± S.E.M. (n = 3). ** $p < 0.01$ versus control. ## $p < 0.01$ versus the same sample without nicardipine. (b) Representative images of the changes in the bioluminescence signal intensity 0, 3, 6, and 9 h after the KCl treatment in the absence

or presence of nicardipine in cultured *Bdnf-Luc* Tg mouse cortical cells. At 5 DIV, luciferin (0.5 mM) was added into the cells, and the bioluminescence time-lapse imaging was performed with LV200 (Olympus). Scale bar = 200 μm. (c) The change in bioluminescence signal intensity in regions of interest (ROI) was analyzed by MetaMorph software (Molecular Devices). Gray lines indicate the change in the signal of each cell, and the black line indicates the average of the signal of all cells (n = 52). Sharp and transient increases in the signals were observed during *in vitro* imaging, which were probably due to cosmic radiations overlapping with the ROI.

Supplementary Figure 3. Visualization of depolarization-induced BDNF induction *in vitro*.

(a) Using the head skin tissue (skin) removed from the Tg mice before *in vivo* imaging and the cerebral cortex (Cx) of the Tg mice after *in vivo* imaging, total RNA was extracted and the expression of endogenous *Bdnf* and *Gapdh* mRNA was examined by RT-PCR. Full-length gels are presented in Supplementary Figure 5. (b) The expression levels of endogenous *Bdnf* mRNA in skin and Cx of the wild-type or the Tg mice were measured by real-time PCR. The level of *Bdnf* mRNA were normalized to that of *Gapdh* mRNA. The data represent the mean ± S.E.M. (n = 3). NS, not significant. (c) The skin tissue isolated from the Tg mice before *in vivo* imaging was put in 50 mM Luciferin solution, and the bioluminescence was measured for 2 min using Clairvivo OPT. (d) The expression levels of *Luc* mRNA in the isolated skin tissues of the wild-type or the Tg mice were measured by real-time PCR. The data represent the mean ± S.E.M. (n = 3).

Supplementary Figure 4. Changes in the bioluminescence signals and expression of endogenous *Bdnf* mRNA in the adipose tissues of aged *Bdnf-Luc* Tg mice with normal and high body weight.

(a) Representative images (dorsal and ventral) of the bioluminescence signal of *Bdnf-Luc* Tg mice (approximately 1-year-old). Scale bar = 20 mm. Body weight of each Tg mouse is also shown. (b) Subcutaneous adipose tissues of each Tg mouse were isolated and total RNA was extracted by TRIsure. Using 1 μg of total RNA, the expression of endogenous *Bdnf* and *Gapdh* mRNA was investigated by RT-PCR analysis. The primer sequences are shown in the Methods section. PCR products were separated on 2% agarose gel. Full-length gels are presented in Supplementary Figure 5.

Supplementary Figure 5. Changes in abdominal bioluminescence signal, endogenous *Bdnf* mRNA and BDNF protein expression in adipose tissue from *Bdnf-Luc* Tg mice.

(a) Representative images of the bioluminescence signal in *Bdnf-Luc* Tg mice after normal or high-fat diets for 2 weeks. Scale bar = 20 mm. After measuring the bioluminescence signal, subcutaneous adipose tissue from each Tg mouse was isolated, and total RNA and total protein were extracted. (b, c) Expression levels of endogenous *Bdnf* mRNA (b) and BDNF protein (c) were examined by real-time PCR and immunoblotting, respectively. Relative band intensities were analyzed using Image J software. The data represent the mean ± S.E.M. (n = 4). * $p < 0.05$ versus normal diet-fed mice. NS, not significant.

Supplementary Figure 6. Full-length gels.

Full-length gels corresponding cropped gels shown in Figs. 1c, 2a, 2b, Supplementary

Figs. 3a, 4b, and 5c.

Supplementary Figure 1

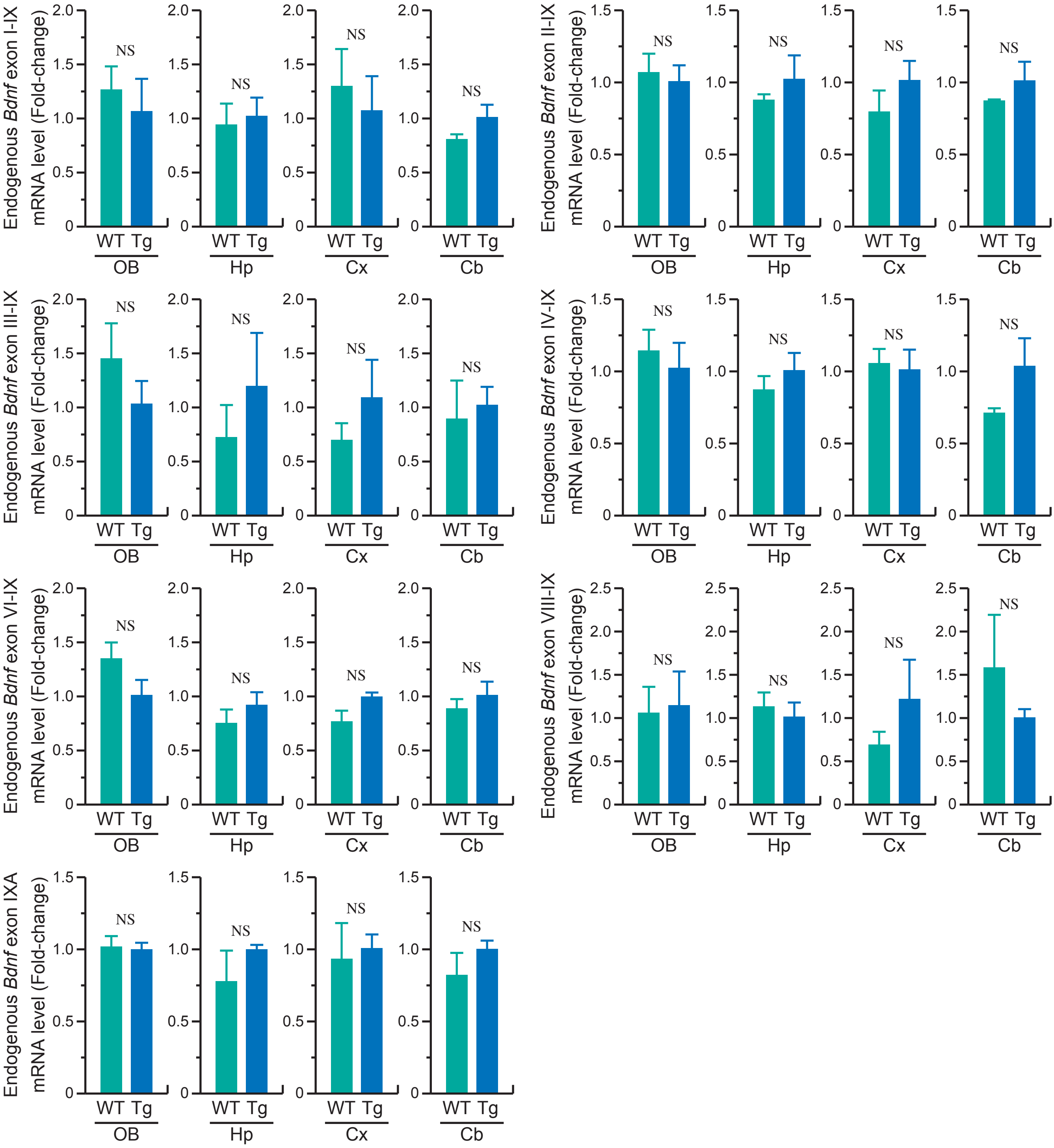

Supplementary Figure 2

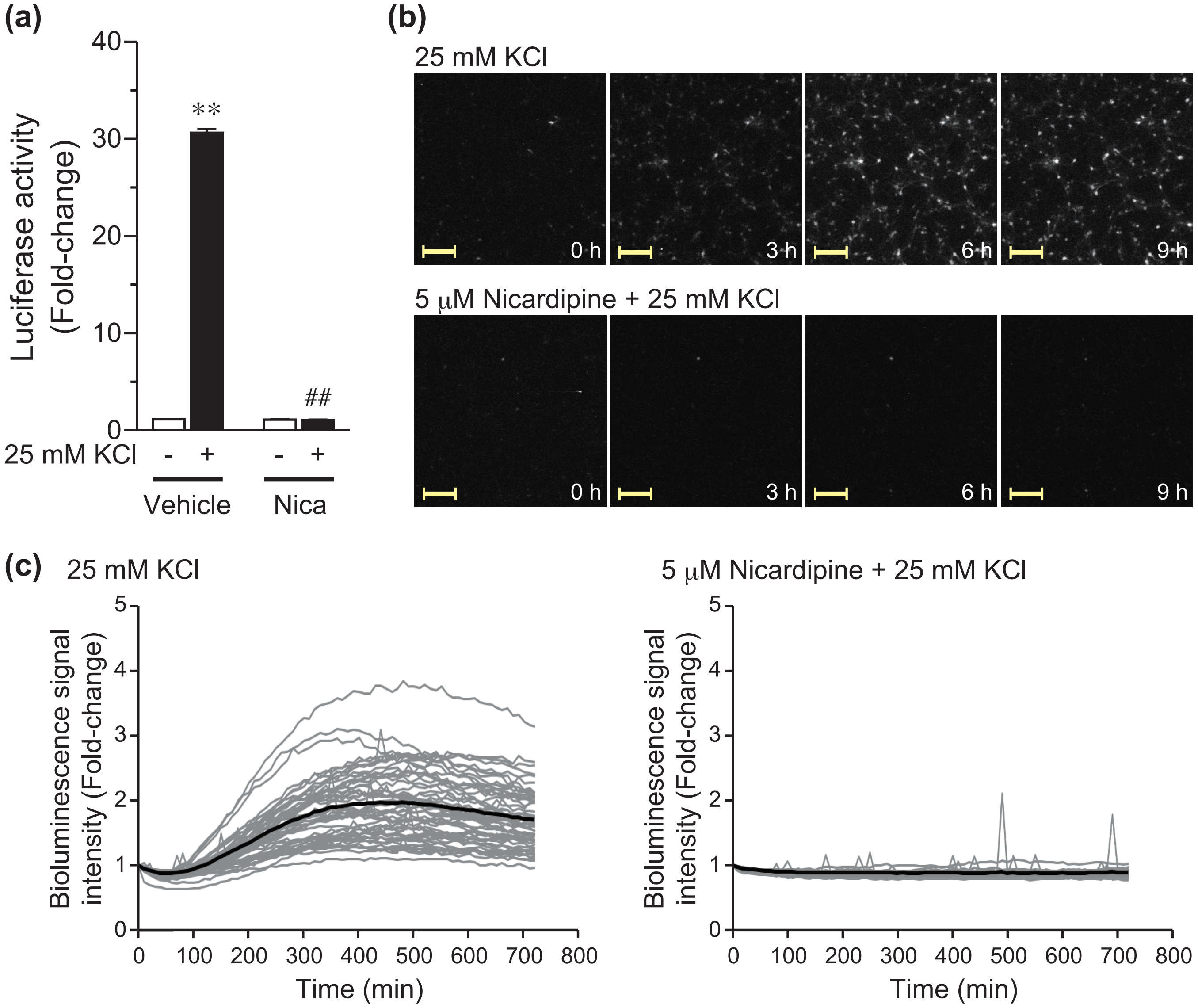

Supplementary Figure 3

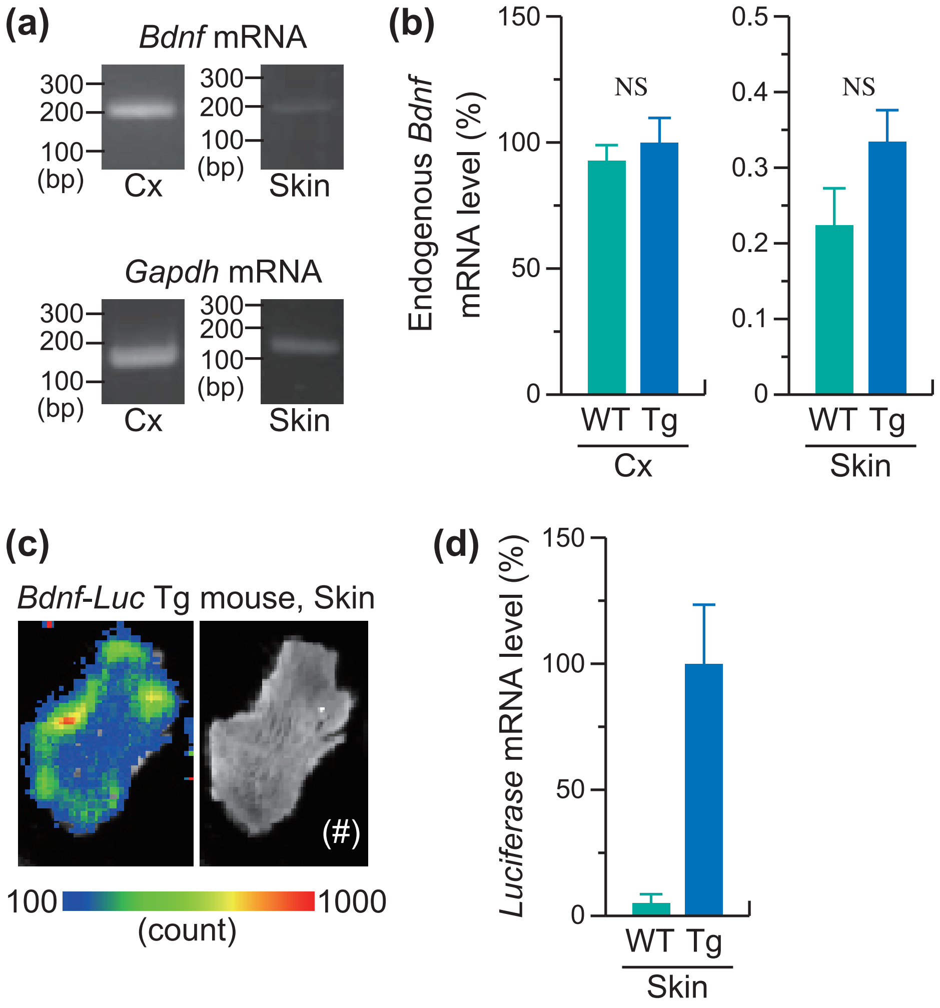

Supplementary Figure 4

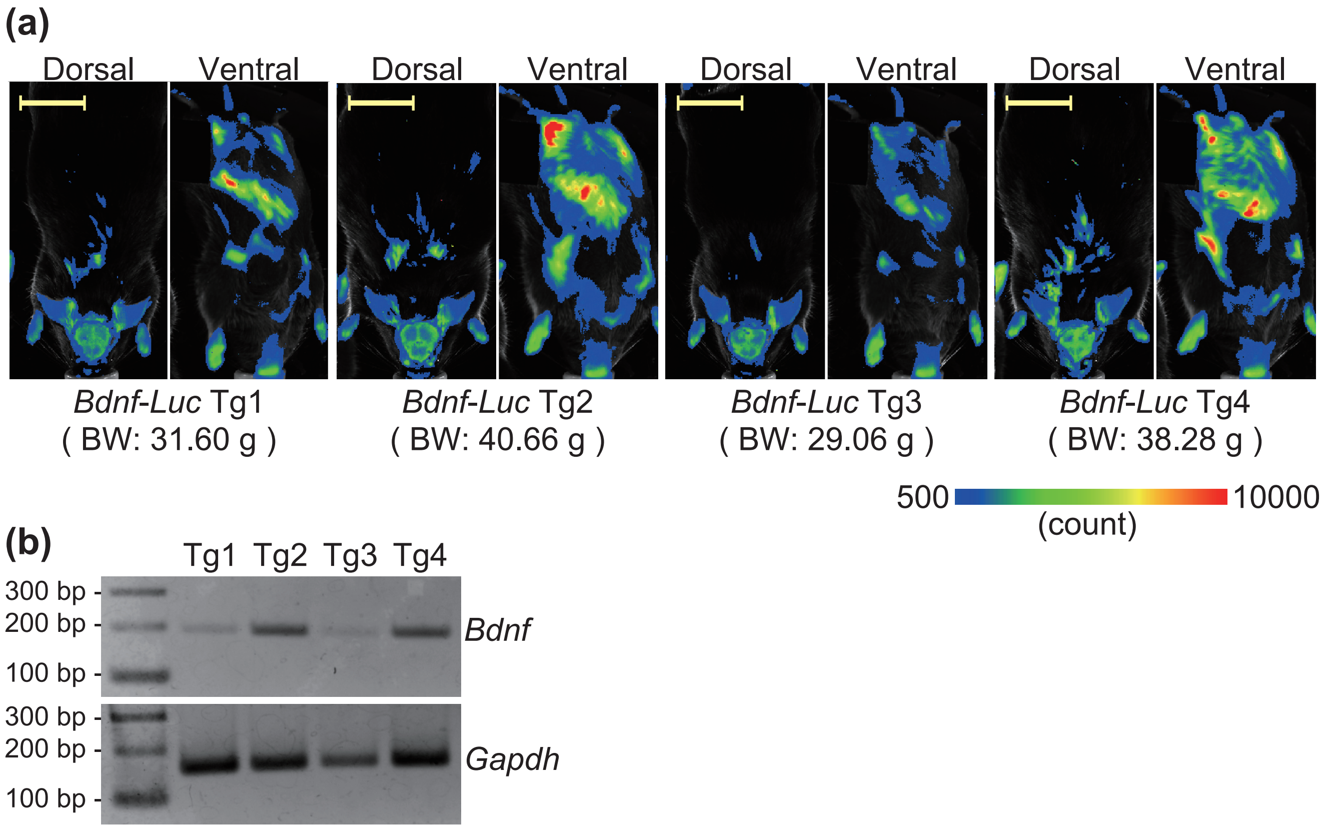

Supplementary Figure 5

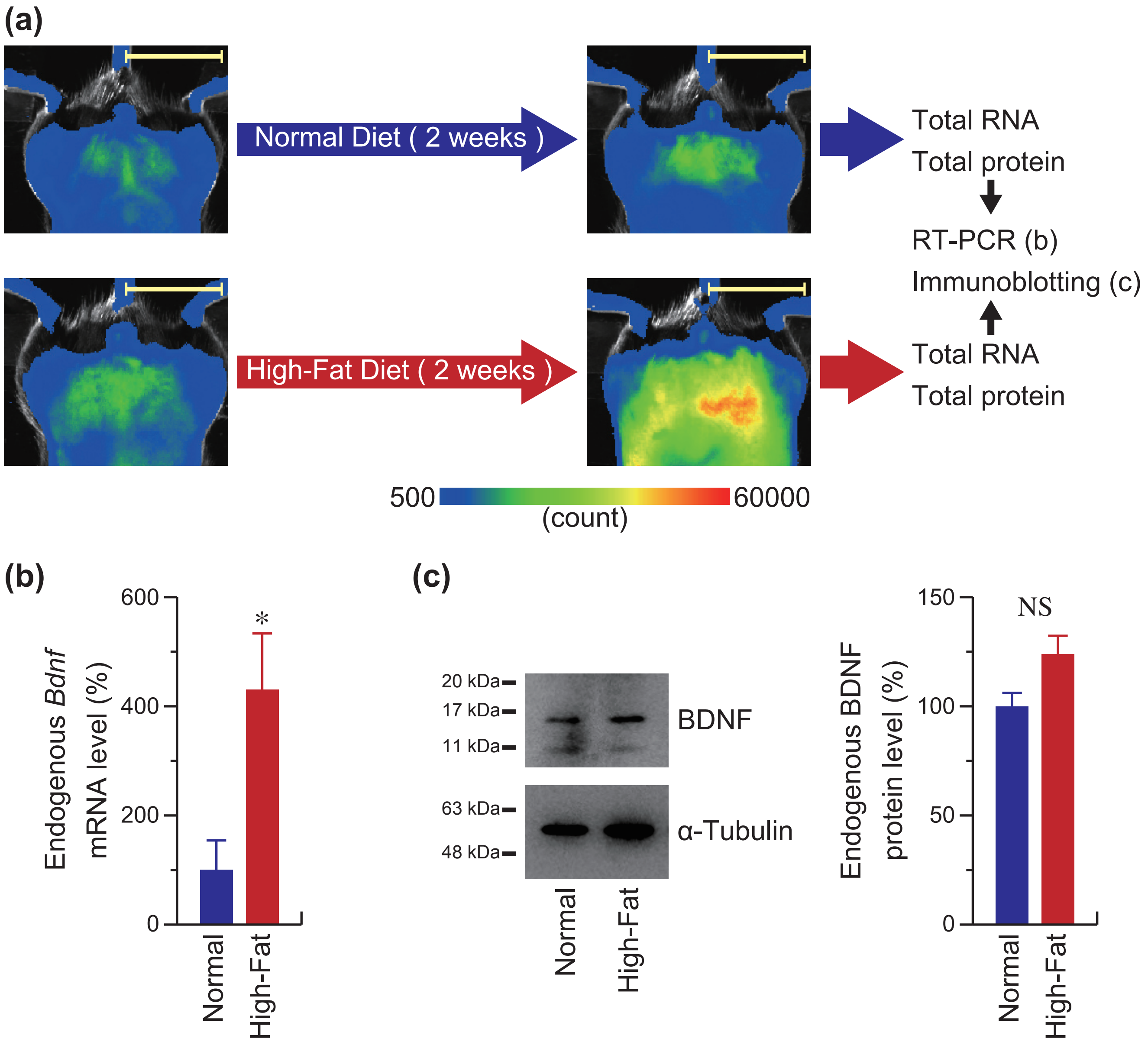

Supplementary Figure 6

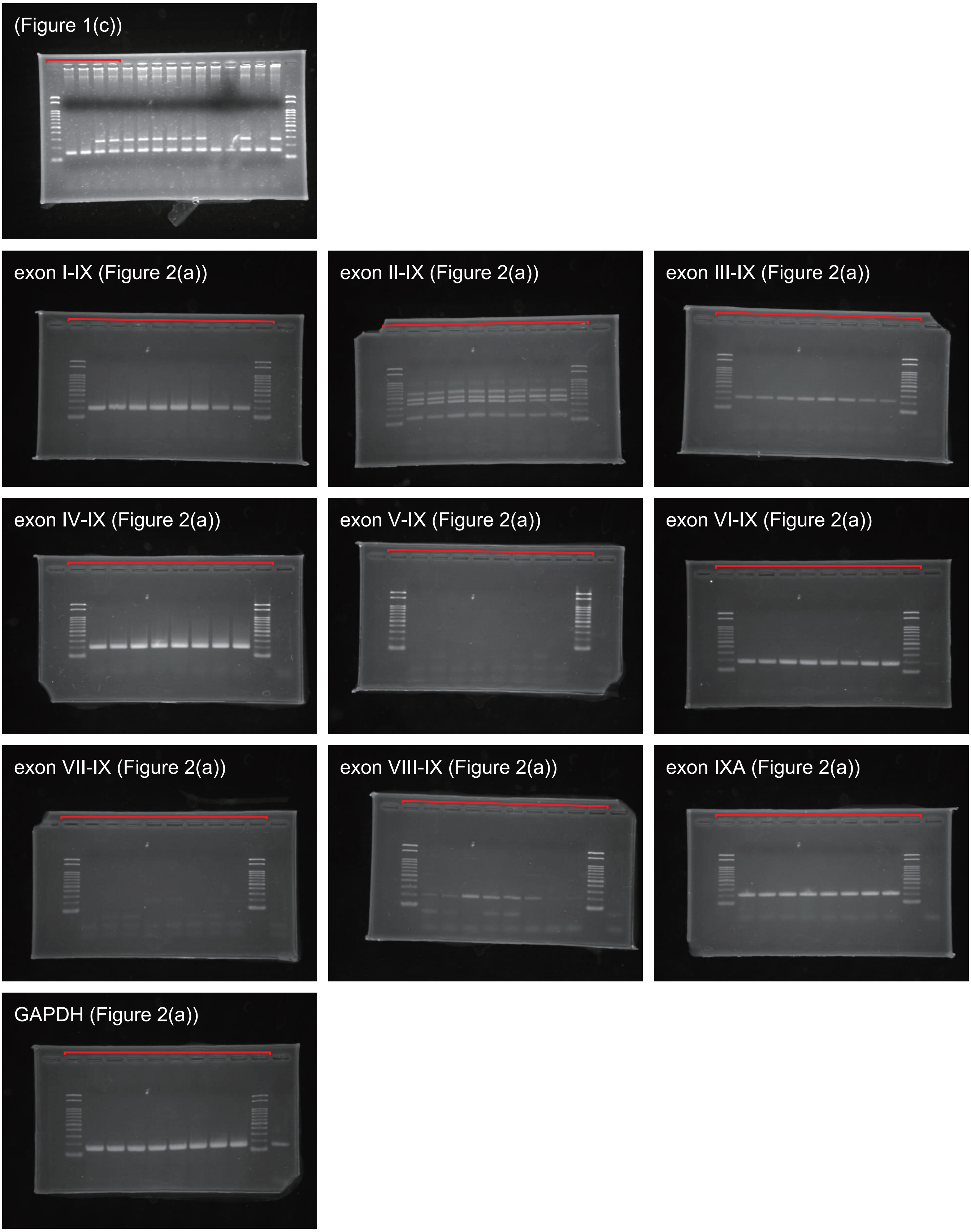

Supplementary Figure 6 (Continued)

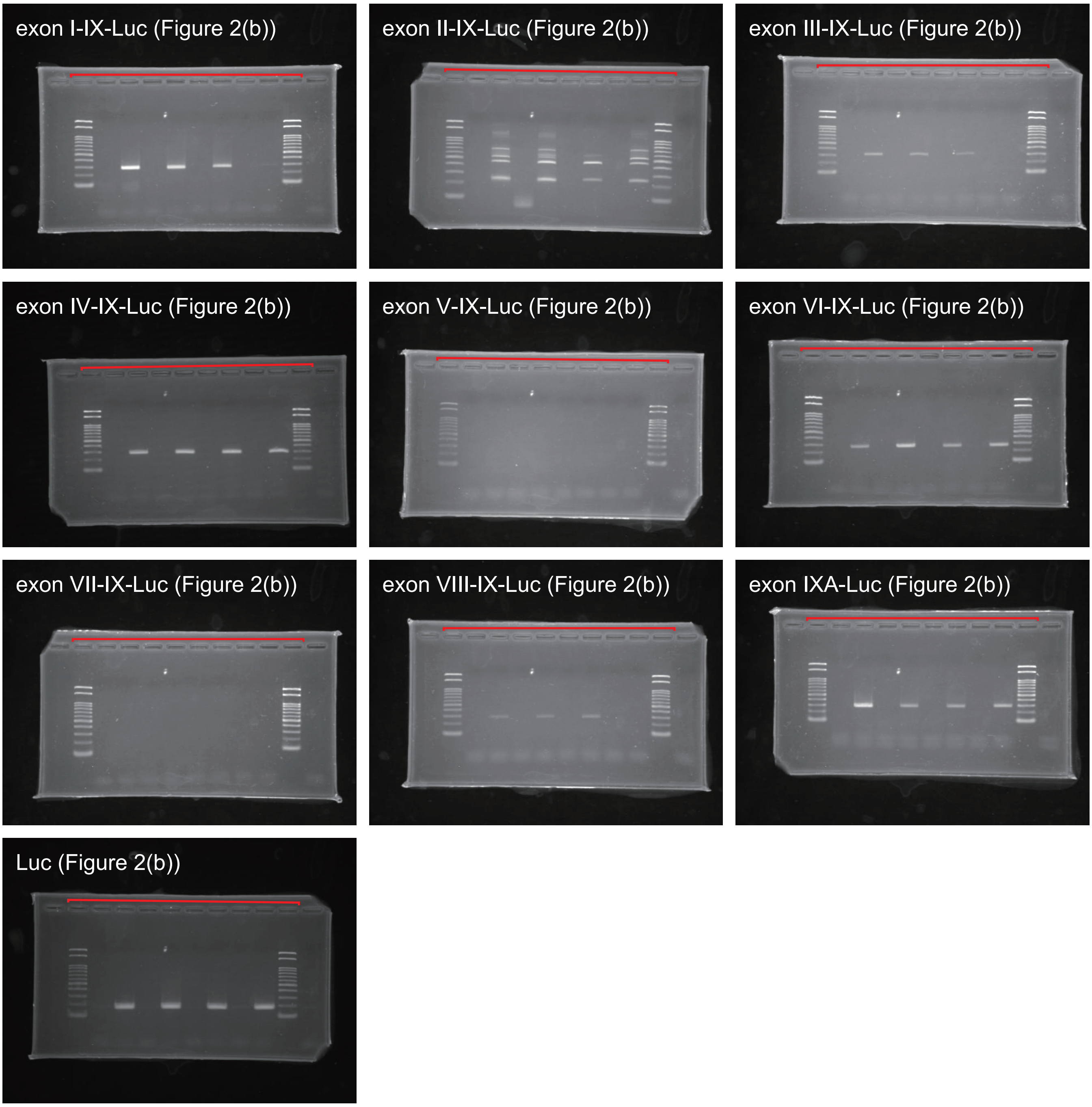

Supplementary Figure 6 (Continued)

Bdnf mRNA
(Supplementary Figure 3(a))

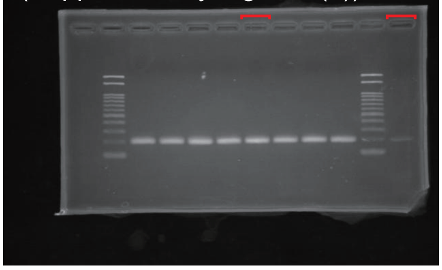

Gapdh mRNA
(Supplementary Figure 3(a))

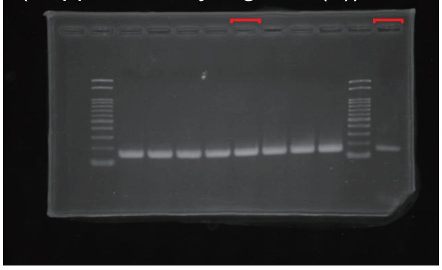

Bdnf (Supplementary Figure 4(b))

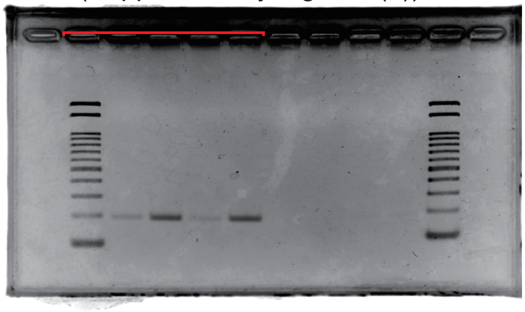

Gapdh (Supplementary Figure 4(b))

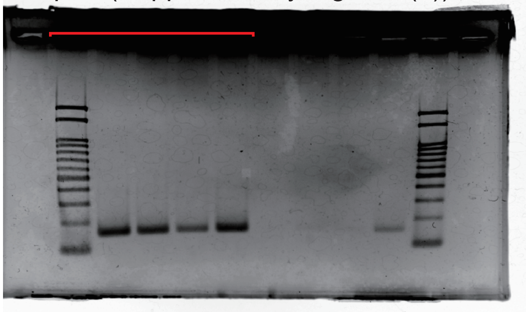

BDNF (Supplementary Figure 5(c))

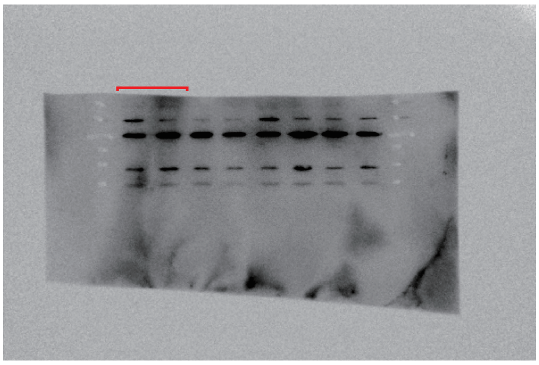

α-Tubulin (Supplementary Figure 5(c))

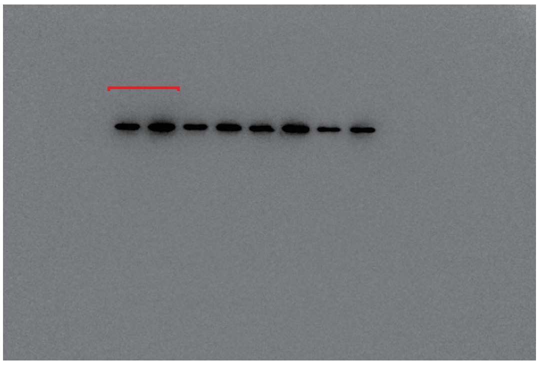

Supplement: Supplementary file 1 — Supplementary information [file 41598_2017_5297_MOESM1_ESM.pdf]
